# Roles of the Hcp family proteins in the pathogenicity of *Salmonella typhimurium* 14028s

**DOI:** 10.1080/21505594.2020.1854538

**Published:** 2020-12-10

**Authors:** Ping Wang, Jun-Fang Dong, Ren-Qing Li, Lei Li, Qing-Hua Zou

**Affiliations:** a Department of Microbiology& Infectious Disease Center, School of Basic Medical Sciences, Peking University Health Science Center, Beijing, China; b Institute for Infectious Disease and Endemic Disease Control, Beijing Center for Disease Prevention and Control, Beijing Research Center for Preventive Medicine, Beijing, China; c The MOH Key Laboratory of Geriatrics, Beijing Hospital, National Center of Gerontology, Beijing, China

**Keywords:** *Salmonella typhimurium*, type VI secretion system(T6SS), hemolysin-coregulatory protein (Hcp), virulence, flagella

## Abstract

The type VI secretion system (T6SS) is a new secretion system that is widely distributed among Gram-negative bacteria. The core component hemolysin-coregulated protein (Hcp) can be used as both its structural protein and secretory protein or chaperone protein. Studies on Hcp are important to elucidate the overall virulence mechanism of T6SS. *Salmonella typhimurium* is an important foodborne pathogen. There are three copies of *hcp* genes identified in *S*. Typhimurium 14028s. This study aimed to characterize the functions of the three Hcp family proteins and to elucidate the interactions among them. The *hcp* gene deletion mutants were constructed by λ Red-based recombination system. Effects of *hcp* mutation on the pathogenicity of 14028s were studied by bacterial competition assays, *Dictyostelium discoideum* assays and mouse model. The three Hcp family proteins were found to play different roles. Hcp1 can affect the transcription of *rpoS* and type 2 flagellar gene and influence the motility of 14028s. It is also involved in the intracellular survival of 14028s in *Dictyostelium discoideum*; Hcp2 is involved in the early proliferative capacity of 14028s in mice and can prevent its excessive proliferation; Hcp3 did not show direct functions in these assays. Hcp1 can interact with Hcp2 and Hcp3. Deletion of one *hcp* gene can result in a transcription level variation in the other two *hcp* genes. Our findings elucidated the functions of the three Hcp family proteins in *S*.Typhimurium and illustrated that there are interactions between different Hcp proteins. This study will be helpful to fully understand how T6SS actions in an organism.

## Introduction

Bacterial pathogens utilize various secretion systems to export toxins or effectors into target cells. There are nine secretion systems discovered so far [1,2]. The type VI secretion system (T6SS) was first described in *Vibrio cholerae* in 2006 [3]. Studies have shown that T6SS plays important role in the pathogenicity of bacteria [4–6]. Currently, there are three different types of T6SS identified [2]. The second type is only found in *Francisella*, the third is only found in *Bacteroides*, and the first type which consists of 13 core genes has been found in most Gram-negative bacteria [2,7]. *S*.Typhimurium is a common pathogen of foodborne diseases worldwide [8,9]. It can be transmitted through the fecal-oral route, and cause diarrhea or other systemic disease by entering the host’s macrophages or dendritic cells [10,11]. Researches on virulence-related genes are necessary to reveal its invasion mechanism.

It has been reported that in *S*.Typhimurium, there is a T6SS gene cluster which was found to play important roles in bacterial competition and in the interaction of bacteria with macrophages or hosts [12–18]. The hemolysin-coregulated protein (Hcp) is an important structural protein which can be stacked into a tube of Hcp hexamers to form the tail of T6SS; moreover, it can also play the roles of secretory protein or chaperone protein [19–23]. The secretion of Hcp is the hallmark of a functional T6SS [24]. Understanding the roles of Hcp is critical to explore the function of T6SS. It is interesting that in some bacteria, more than one copy of *hcp* genes was found. However, even if multiple copies of *hcp* genes exist, their functions are not redundant. They can take different roles in bacterial competition, interaction with macrophages or involve in the biological phenotypes such as the motility or biofilm formation of bacteria [25–29]. However, it remains a question that how the Hcp family proteins function? Are there interactions among them? In *S*.Typhimurium 14028s, we found three copies of *hcp* genes (Additional file 1: Figure S1), two of them (*hcp1* and *hcp2*) are on the *Salmonella* pathogenic island 6 (SPI-6). The proteins encoded by the two genes are very similar, with only 10 amino acid differences between them [23]. There is also an orphan *hcp3* gene not linked with SPI-6 [12]. Hcp3 only shared 26%-27% similarity with Hcp1 and Hcp2 and it is more homologous to the Hcp of *S.bongori* and *Pseudomonas* [12]. Previous studies have shown that the expression of Hcp1 is enhanced in bile salt environment and it can interact with amidase Tae4 to exert antibacterial activity [5]. In this study, we constructed *hcp* mutants and investigated the roles of the three Hcp family proteins by different assays. We further investigated the interactions between different Hcp proteins to elucidate the mechanism behind them.

## Results

### Effects of hcp mutations on the growth rates, antimicrobial resistance, biofilm formation, and the motility of S.Typhimurium14028s

**Figure 1.**
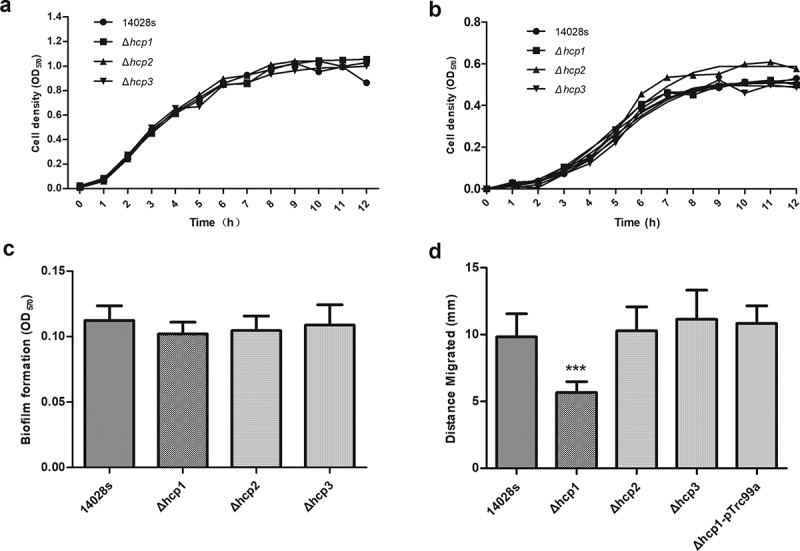
Growth characters in LB (a) and M9-glucose minimum medium (b), biofilm formation ability (c) and motility (d) of wild type and mutant strains. Motility of the wild type and mutant strains was determined by measuring the diameter of the column. Error bars represent the SDs. Significant differences were defined by *P* < 0.05 (*), *P* < 0.01 (**), and *P* < 0.001 (***) compared to the wild type strain 14028s

All mutant strains were confirmed with PCR using specific primers. To investigate whether *hcp* knock-out would affect the growth of *Salmonella*, we tested the growth rates of the Δ*hcp* mutants and wild type 14028s in both LB and M9-glucose minimum medium. As shown in Figure 1(a,b), there were no significant differences among them, indicating that knocking out *hcp* did not affect the growth of *Salmonella*. A recent study found that deletion of the T6SS core component can influence the antimicrobial resistance of *A. baumannii* [30]. In this study, we tested the effects of *hcp* mutation on the antimicrobial resistance of *Salmonella* with five antibiotics, ampicillin, gentamicin, streptomycin, naphthalidine, and tetracycline. As shown in Table 1, the MIC of the wild type and mutants for the five antibiotics were 16 μg/mL, 2 μg/mL, 32 μg/mL, 4 μg/mL, and 2 μg/mL, respectively. There were no significant differences between wild type and mutant strains. The same result was obtained using the K-B method (Additional file 2: Figure S2). Compared with the wild type strain 14028s, there was a mild decrease tendency in the biofilm formation ability of Δ*hcp1* and Δ*hcp2* mutants (Figure 1(c)). However, there were no significant differences between them.

**Table 1.** Effects of *hcp* mutations on the MICs of antimicrobials

Strains	Ampicillin	Gentamicin	Streptomycin	Naphthalidine	Tetracycline
14028s	16	2	32	4	2
Δ*hcp*1	16	2	32	4	2
Δ*hcp*2	16	2	32	4	2
Δ*hcp*3	16	2	32	4	2

The motility of wild type and mutant strains was examined by measuring the diameter of the circles on the swimming plate. As shown in Figure 1(d), the motility of the Δ*hcp1* mutant was significantly decreased (P < 0.001). Complementation of *hcp1* restored the motility of Δ*hcp1* mutant. In contrast, deletion of *hcp2* and *hcp3* did not affect the motility of the strain.

### Effects of hcp mutations on the bacterial competition ability

**Figure 2.**
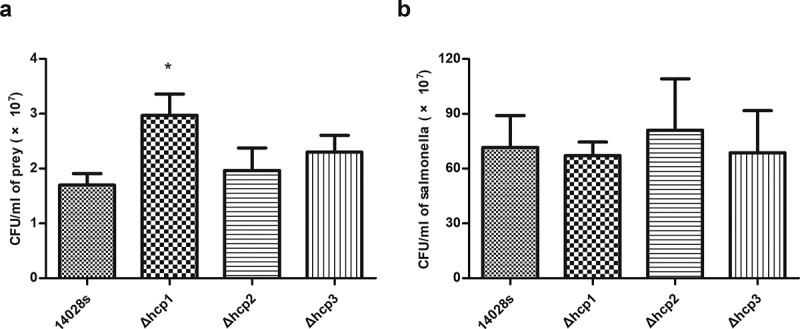
Results of bacterial competition assay. The surviving prey (*E.coli* JM109) (a) and the surviving salmonella strains (b) after 12 h co-incubation on LB plate supplemented with 0.05% porcine bile salts were measured by counting CFU. Error bars represent the SDs. Significant differences were defined by *P* < 0.05 (*), *P* < 0.01 (**), and *P* < 0.001 (***) compared to the wild type strain 14028s

Previous studies revealed that the bacterial competition ability of *S*.Typhimurium SL1344 to *E. coli* was significantly reduced after knocking out *hcp1*, and knocking out *hcp2* has no effect on bacterial competition [5]. This study focused on whether *hcp3* played a role in bacterial competition. Our results showed that consistent with previous study, mutation of *hcp1* significantly affected the bacterial competition ability of 14028s (P < 0.05) and mutation of *hcp2* showed no significant effect. The competition ability of Δ*hcp3* was similar with that of the wild type strain, suggesting that *hcp3* was not implicated in *Salmonella* bacterial competition (Figure 2(a)). The CFUs of *Salmonella* strains after competition showed no significant statistical difference (Figure 2(b)).

### Effects of hcp mutations on intracellular survival of Salmonella in D.discoiseum

**Figure 3.**
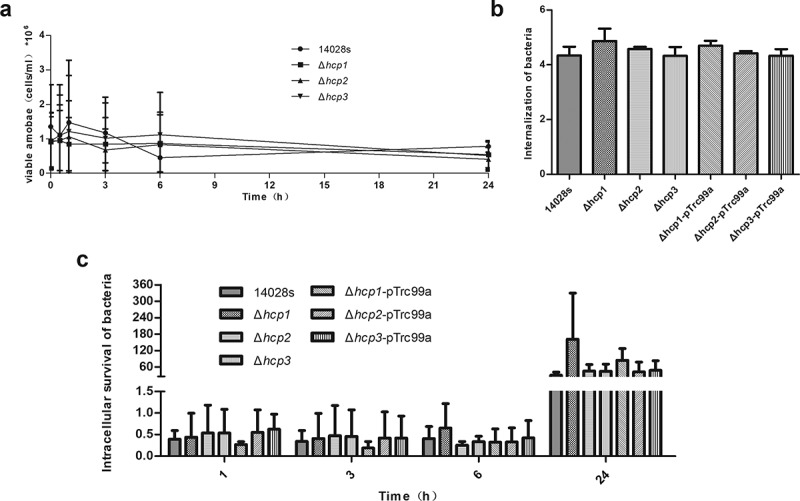
The population of viable amoebae at each time point when incubated with the wild type and mutant strains (a) were tested by Trypan blue exclusion and counting on a Neubauer chamber. The internalization of the wild type and mutant strains in *D.discoideum* (b) were calculated as CFU_t=0_/CFU_inoculum_ and converted negative logarithmically. The intracellular survival of the wild type and mutant strains in *D.discoideum* (c) were calculated at each time point as CFU_t=x_/CFU_t=0_. Error bars represent the SDs. Significant differences were defined by *P* < 0.05 (*), *P* < 0.01 (**), and *P* < 0.001 (***) compared to the wild type strain 14028s

*Dictyostelium discoideum* is an amoeba model which was widely used for host–pathogen interactions [3]. It was also shown that T6SS was required for S. Typhimurium to survive intracellularly in the social amoeba *Dictyostelium discoideum* [15]. To determine the roles of the three Hcp family proteins in the intracellular survival of *Salmonella*, we co-incubated both wild type 14028s and its mutants in *D.discoiseum* and let *D.discoiseum* fed on bacteria. There were no significant differences between the number of viable *D.discoideum* when incubated with the wild type 14028s and *hcp* mutants (Figure 3(a)). So the influence of the number of viable *D.discoiseum* can be excluded. Then the internalization and intracellular survival of *Salmonella* in *D.discoideum* were tested. It was found that all the mutants showed an internalization level similar to that of the wild type14028s (Figure 3(b)). For the intracellular survival ability, there were no significant differences for all the strains within 3 h; however, Δ*hcp1* showed a strong increasing trend at 6 h and 24 h, although there were no statistically significant differences. Complementation of *hcp1* can suppress the increasing trend. These results indicated that deletion of *hcp1* can result in increased intracellular survival ability in *D.discoideum* (Figure 3(c)).

### Virulence in mouse model of systemic infection

**Figure 4.**
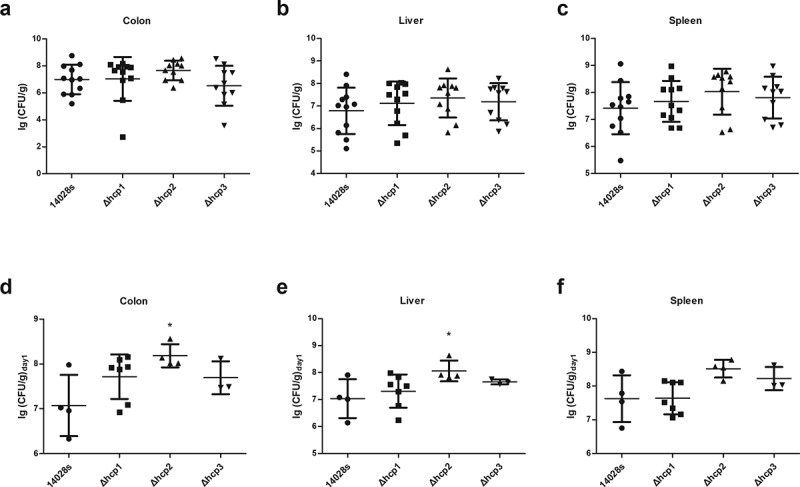
Virulence of the wild type and mutant strains in mouse model of systemic infection were determined by examining the bacteria concentrations in different organs (colon, spleen, and liver)

To study the roles of the three Hcp family proteins played in the virulence to mouse model, we challenged BALB/c mice with two different *Salmonella* levels. At first, we challenged mice with a low level (200 μL bacterial suspensions normalized to 10^6^ CFU/mL) which is not lethal to mice and examined the proliferation of bacteria every day. We found that the weight of mice spleen and the number of the bacteria recovered from the spleen of the Δ*hcp*1 and Δ*hcp*3 group showed no significant difference with wild type 14028s group, in contrast, the Δ*hcp*2 mutant group showed an increasing tendency compared with wild type 14028s group within 2 days, but on day 5 the difference between the two groups disappeared (Additional file 3: Figure S3), suggesting Hcp2 may be involved in the early proliferative capacity of 14028s in mice. To verify this, we challenged mice with a higher level of *Salmonella* (200 μL bacterial suspensions normalized to 10^8^ CFU/mL). Our results showed that there were no significant differences in the survival rate among different groups (Additional file 4: Figure S4). However, when calculating the number of *Salmonella* in the mice that died on the first day (within 24 h after inoculation), we found that the Δ*hcp2* mutant showed a significant higher colonization ability in the colon and liver (p < 0.05), and there was also a higher trend in the spleen (Figure 4(d–f)). The other mutants showed no significant differences with the wild type strain. When we examined the number of bacteria on day 5, there were no significant differences between wild type 14028s and the mutants, which is consistent with the results of low-level inoculation (Additional file 3: Figure S3b).

(a–c) Colonization ability in different organs of mice. (d–f) Colonization ability in different organs of mice that was dead within 24 h after inoculation. Error bars represent the SDs. Significant differences were defined by *P* < 0.05 (*), *P* < 0.01 (**), and *P* < 0.001 (***) compared to the wild type strain 14028s.

### Interactions of the three Hcp family proteins

**Figure 5.**
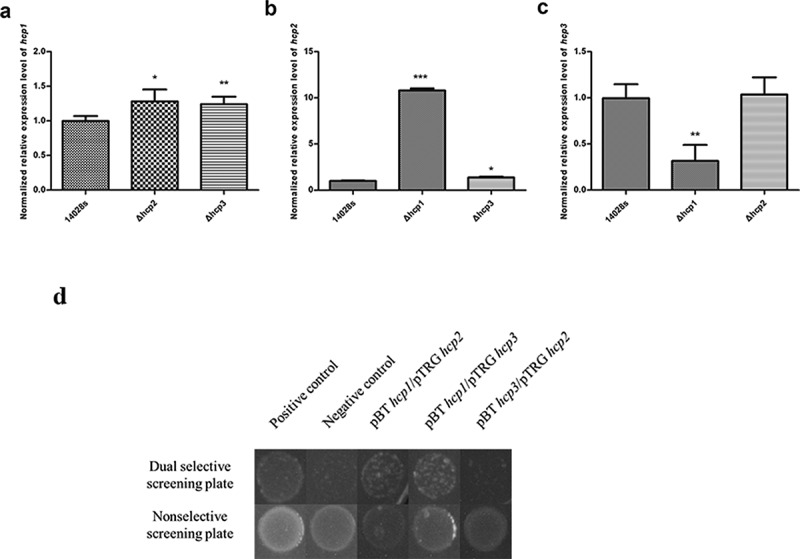
Interactions of the three Hcps were tested by qRT-PCR and bacteria two-hybrid assay. (a–c) The normalized expression level (2^−ΔΔct^) of *hcp*s of the wild type and mutant strains. 16S rRNA was used as the internal parameter. Error bars represent the SDs. Significant differences were defined by *P* < 0.05 (*), *P* < 0.01 (**), and *P* < 0.001 (***) compared to the wild type strain 14028s. (d) Bacteria two-hybrid analyses. Different strains that have different plasmid combinations were spotted on dual selective screening plate and nonselective screening plate respectively at 30°C for 24 h. The strains can grow on the dual selective screening plate if the proteins on the plasmids can interact. The experiments were done at least in triplicate, and a representative result is shown

Since there are some similarities among the sequences of the three Hcp family proteins, we tried to see whether there are interactions between them. The *hcp* transcription levels of the wild type 14028s and *hcp* mutant strains were tested by qRT-PCR. It was found that the *hcp2* transcription level in the Δ*hcp1* mutant was significantly increased (P < 0.001), and the *hcp3* transcription level was significantly decreased (P < 0.01) (Figure 5(a)). For the Δ*hcp2* mutant, the *hcp1* transcription level was significantly increased (P < 0.05), while the *hcp3* transcription had no difference (Figure 5(b)). The *hcp1* transcription level of Δ*hcp3* mutant was significantly increased (P < 0.01), and the *hcp2* transcription level was also increased (P < 0.05) (Figure 5(c)).We then tested whether there are interactions among the Hcp proteins. The results of bacterial two-hybrid assay indicated that Hcp1 can interact with Hcp2 and Hcp3, while Hcp2 and Hcp3 cannot interact with each other (Figure 5(d)).

### Effects of hcp mutations on the transcription of rpoS, fimH and flagellar genes

**Figure 6.**
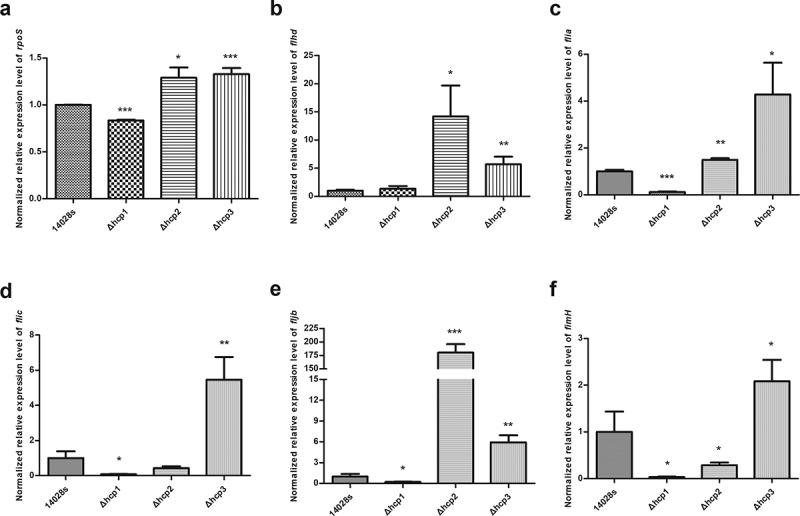
The normalized expression level (2^−ΔΔct^) of *rpoS*(a), flagellar genes *flhd* (b), *flia* (c), *flic* (d), *fljb* (e), and *fimH* (f) of the wild type and mutant strains were tested by qRT-PCR. 16S rRNA was used as the internal parameter. Error bars represent the SDs. Significant differences were defined by *P* < 0.05 (*), *P* < 0.01 (**), and *P* < 0.001 (***) compared to the wild type strain 14028s

Lastly, we tested the transcription levels of *rpoS, fimH* and flagellar genes in the wild type 14028s and the *hcp* mutants. Since our results showed that mutation of *hcp1* can influence the motility of *Salmonella*, we tested the transcription levels of the *rpoS, fimH* and flagellar genes to elucidate the mechanism behind it. Results (Figure 6) showed that in the Δ*hcp1* mutant, the transcription of *rpoS, flia, fljb, flic, and fimH* was significantly decreased. In the Δ*hcp2* mutant, the transcription of *rpoS, flhd, flia* and *fljb* was significantly increased and the transcription of *fimH* was significantly decreased. In the Δ*hcp3* mutant, the transcription of *rpoS, flia, flhd, fljb, flic, and fimH* were increased significantly.

## Discussion

In this study, we constructed *hcp* mutants and tested the effects of *hcp* mutations on a variety of characters in *S*.Typhimurium 14028s. Our results showed that *hcp* mutation had no significant effect on the growth of 14028s. So the subsequent results can exclude the influence that may be caused by growth ability. Previous study showed that mutation of T6SS core genes can influence the antimicrobial resistance in *A.baumannii* [30]. *S*.Typhimurium was found to have a high resistant rate to streptomycin, ampicillin, nalidixic acid, chloramphenicol, gentamicin, and tetracycline [31]. In this study, we tested the resistance of the *hcp* mutants to these antibiotics, and the results showed that *hcp* mutations of 14028s had no significant effect on the antimicrobial resistance. In *Aeromonas hydrophila*, it was found that *hcp* was implicated in the biofilm formation ability [28]. However, in our study, we found that *hcp* mutation showed no effect on biofilm formation.

The assembly and functional expression of flagella involves three types of genes which were expressed in a cascade. *flhD* is a representative of type 1 flagellar gene which is necessary for the activation and transcription of type 2 gene promoters. *fliA* belongs to the type 2 flagellar gene, which encode proteins that constitute the flagellar secretion device and participate in the transcription of the type 3 flagellar genes which participate in the final motility function of bacteria [32]. In this study, we found that deletion of *hcp1* led to significant defects in the motility of *S*.Typhimurium. This can be explained by the data from qRT-PCR, which showed that in Δ*hcp1* mutant the type 2 flagellar gene *fliA* and type 3 flagellar genes *fliC* and *fljB* all showed significant decreased transcriptions, which may result in reduced synthesis of flagellin and thus influence the motility. Previous studies [33,34] have found that *rpoS* can regulate the expression of T6SS to help bacteria survive under stress conditions, another study [35] also found that it can be regulated by T6SS. In this study, we found the deletion of *hcp* can affect the transcription of *rpoS*. Since *rpoS* has also been found to be involved in the formation of flagella [33], our study indicated that Hcp can modulate the expression of *rpoS* to regulate the expression of flagella genes and thus affect the motility of bacteria.

T6SS was required for *S*. Typhimurium to survive intracellularly in the social amoeba *Dictyostelium discoideum* [15,36]. However, the mechanism and the effectors associated with this process remain unknown. Some studies revealed that although flagellar mutants are more difficult to invade epithelial cells, mutants have stronger virulence to mice and a faster net growth in macrophages within 4–24 h [32,37]. In addition, in the study of avian pathogenic *Escherichia coli* (APEC), it was found that deletion of *hcp* gene significantly affected the expression of fimbriae subunit protein and decreased the adhesion and invasion of cultured cells in vitro [38]. Consistent with these results, our data showed that the transcription of fimbriae gene *fimH* was significantly decreased in the Δ*hcp1* and Δ*hcp2* mutants. And we found that although it was not statistically significant, the internalization level of Δ*hcp1* and Δ*hcp2* was lower than that of the wild type strain 14028s. The intracellular survival of Δ*hcp1* showed a strong increasing trend at 6 h and 24 h. We postulated that the down-regulation of the flagellar gene caused by the deletion of *hcp1* makes it impossible for *Salmonella* to synthesize flagella and thus reduce the metabolic burden, which may be one of the main reasons of increased intracellular survival in amoeba.

In order to survive in a multi-bacterial environment such as the host gut, bacteria need to compete with each other [39]. Sana et al. found that *hcp1* can affect the ability of *S*.Typhimurium to participate in bacterial competition with bile salts, while *hcp2* has no effect [5]. The same results were obtained in our experiments. Besides this, we found Δ*hcp3* mutant had the same competitive ability as the wild type strain. Therefore, Hcp1 is mainly involved in bacteria competition and the other two Hcps showed no direct effects.

By analyzing the transcription levels and protein interactions, we found there were indeed mutual interactions between Hcp proteins. There was a balance between Hcp1 and Hcp2, and they may complement each other due to the similarity in their amino acid sequences. The transcription of *hcp3* decreased significantly in the Δ*hcp1* mutant, we suppose that Hcp3 may mainly assist the function of Hcp1. We hypothesized that *hcp3* might function more as a secretory protein. Further studies were needed to verify these assumptions.

In our animal model assay, it was found that the colonization ability of Δ*hcp2* mutant in the liver and colon increased significantly within 24 h after inoculation. It has been reported that deletion of *pcgL* and *scis* can also increase the proliferation of *Salmonella* in the liver and spleen in mice and in macrophages [40,41]. There is a symbiotic view that bacteria may deliberately reduce their virulence to allow themselves to continue to spread [42]. Gene expression in the host cell will cause a difference in the cellular environment. Some environments will promote cell proliferation, which is conducive to immune evasion and the establishment of niche, while other environments will allow bacteria to reproduce [43]. A study showed that the deletion of T3SS-related genes leads to increased intracellular reproduction and reduced spread [44]. In addition, there is one hypothesis that *Salmonella* can use the bacteria competition ability mediated by T6SS to clear its niche [39]. We hypothesize that Hcp2 may just play this balance function or elimination mechanism to escape the host’s killing.

In summary, we conducted a comprehensive study and concluded that the three Hcp family proteins play different roles in *S*.Typhimurium14028s, and the three Hcp family proteins can interact and balance with each other. Hcp1 plays an important role in bacterial competition, long-term intracellular survival in eukaryotic cells, and bacterial motility. These effects may be due to the expression of flagellar genes. Hcp2 mainly contributes to the early stage of bacterial infection in mice. Hcp3 mainly assists the function of Hcp1. We assume that under different conditions, bacteria may selectively use different Hcp proteins to form hexamer pipes, but whether such pipes are a single Hcp stack, or whether two or three Hcp proteins together still need further research. Researches on those virulence-related genes are necessary to reveal the invasion mechanism of the bacteria.

## Materials and methods

### Bacteria strains and cultural condition

Bacteria and plasmids used in this study were listed in Table S1 (Additional file 5) and Table S2 (Additional file 6). Bacteria were grown in LB broth unless otherwise stated. Antibiotics were added when necessary. The concentrations of antibiotics used in this study were: kanamycin: 50 mg/L; ampicillin: 100 mg/L; chloramphenicol: 25 mg/L; gentamicin: 10 mg/L; tetracycline: 15 mg/L.

The eukaryotic cell model used in this study was *Dictyostelium discoideum* strain AX4, which was obtained from the Dicty Stock Center and revived with standard protocols [45]. Briefly, solid culture was carried out by plating on SM media or LPB media containing *E. coli* B/r, and liquid culture was carried out by aseptic culture in HL5 liquid media.

### Construction of mutant and complement strains and determination of growth characters

The *hcp* mutant strains were constructed using the λ Red-based recombination system described in previous study [46]. Briefly, the fragment containing a 56 bp homologous fragment of target gene and kanamycin resistance gene which comes from pKD4 was introduced into 14028s containing pKD46. After recombination, the pKD46 was removed by cultured at 42°C. Then, pCP20 was introduced to obtain a pure gene knockout strain without kanamycin resistance. pTrc99a integrating the target genes were introduced into the mutant strains to produce complement strains.

To verify whether *hcp* mutation affects the growth ability of 14028s, the growth characters were measured: bacteria were cultured overnight and adjusted to OD_600_ ~ 1, then diluted 1:100 to fresh LB broth or M9-glucose (0.2%) minimum medium [47] and cultured with shaking, then 200 μL was added to 96-well plate. The growth rates were detected every hour by measuring the value of OD_570_.

### Biofilm assays

Single colonies were picked from LB Agar, transferred to fresh biofilm induction medium YP media (2% glucose, 2% peptone, 1% yeast extract), and cultured overnight at 30°C with shaking. The bacteria solutions were adjusted to OD_600_ ~ 1 and inoculated in fresh media with a rate of 1:100, and then 200 μL of the culture were added to each well of 96-well plate and incubated at 30°C for 72 h. Then the supernatant and the unattached bacteria were gently removed and washed away with distilled water. The bioflim was stained with 200 μL of 2% crystal violet dye solution for 20 min, and washed three times with water. Then, 200 μL of 95% ethanol was added to dissolve the crystal violet. After 20 min, the biofilm formation ability was determined by measuring the value of OD_570_.

### Antimicrobial susceptibility assays

The antimicrobial susceptibility assays were performed using the MIC broth dilution method for ampicillin, gentamicin, nalidixic acid, streptomycin, and tetracycline. Single colonies of bacteria were inoculated in LB broth and cultured overnight at 37°C, and then the cultures were adjusted to OD_600_ ~ 0.5, and transferred to fresh MH medium at a ratio of 1:100. At last 100 μL of the culture was added to 96-well plate and cultured at 37°C. After 16–20 h, the OD_570_ were detected by microplate reader to determine the minimum inhibitory concentration (MICs). In addition, we used K-B method to determine the drug susceptibility of ampicillin, gentamicin, streptomycin, and tetracycline again according to the national clinical test operating protocol.

### Motility assays

Single colonies were inoculated in LB broth, and the overnight culture were normalized to OD_600_ ~ 1; then, 1 μL of the bacteria suspensions were spotted on the center of swimming plate (1% tryptone, 0.25% Sodium chloride, 0.3% agar) respectively. After 18 h incubation, the migrated distances of the colonies were measured.

### Bacterial competition assays

Bacterial competition assays were performed according to previous studies [5]. Overnight cultures were inoculated into fresh LB broth at a ratio of 50:1. After 4 h, the bacteria were washed with PBS and re-suspended to a concentration of approximately 10^10^ CFU/mL. *Salmonella* and *E.coli* were mixed at a ratio of 10:1 and then co-cultured on LB agar containing 0.05% porcine bile salts at 37°C for 12 h. The bacteria were washed and re-suspended with PBS, and then the colony-forming units (CFU) were determined by plating serial dilutions on LB agar plates with appropriate antibiotics.

### Dictyostelium invasion assay

*Dictyostelium* invasion assay was performed as described in previous literature with minor modifications [15]. Briefly, the *Dictyostelium* AX4 was adjusted to a concentration of 5*10^5^ cells/mL, and 2 mL of the suspension was pipetted into a 6-well plate and grown at 22°C for 1 hour to let it adhere to the surface. Then, AX4 was co-incubated with wild type or mutant strains in HL5 media using a multiplicity of infection (MOI) of 100 bacteria/amoeba for 1 h. The *Dictyostelium* were washed three times with Soerensen buffer and then re-suspended in 2 mL HL5 media (t = 0) and incubated at 22°C for different time points. At the time point 0, 1, 3, 6, 24 h, the infected cells were washed with Soerensen buffer containing 10 mg/L gentamycin. Then the *Dictyostelium* were washed with Soerensen buffer to remove residual antibiotics, and lysed with 0.2% TritonX-100. The colony-forming units (CFU) were determined by plating serial dilutions on LB agar plates with appropriate antibiotics. The internalization was calculated as CFU_t=0_/CFU_inoculum_ and expressed as – log_10_. The survival ability of each time point was calculated as CFU_t=x_/CFU_t=0_. Viable amoeba were determined by Trypan blue dying and counted on Neubauer chamber at each time point.

### Mice infection assays

Mice infection assays were performed following the Guide for the Care and Use of Laboratory Animals, National Research Council, 1996, and were approved by the ethics committee of Peking University Health Science Center of China. 6–8 week old female BALB/c mice were randomly assigned to five groups and housed under specific pathogen-free condition with sterile water and food. The mice were starved for 16 h before intra-gastrically administered with 200 μL of 5% sodium bicarbonate. After 30 minutes, 200 μL bacterial suspensions normalized to 10^6^ CFU/mL or 10^8^ CFU/mL were inoculated. Once a mouse was dead, it was removed quickly from the cage, dissected and the liver, spleen, and colon were picked for culture. The organs were weighed, homogenized, and cultured on MacConkey agar plate to determine the CFU. On day five, mice were sacrificed with cervical dislocation and their organs were removed for culture to calculate the CFU.

### RNA extraction and real-time quantitative polymerase chain reaction (qRT-PCR)

Bacteria were cultured overnight, and RNA extraction was performed using RNAprep Pure Kit (TianGen). The extracted RNA was reversed to cDNA using the All-in-One^TM^First-Strand cDNA Synthesis Kit (TaKara). Then, qRT-PCR was performed using the 2*SYBR Green qPCR Master Mix (Low Rox). The CT value was obtained by using the 7500 Fast DX instrument, the ΔCT value was obtained by subtracting from the internal parameter (16S rRNA), and the normalized relative expression level of the target genes was calculated by the comparative cycle threshold (2^−ΔΔCT^).

### Bacteria two-hybrid assay

BacterioMatch® II Two-Hybrid system (Stratagene, La Jolla, CA, USA) was used for bacteria two-hybrid assay. The *hcp* fragments were cloned into pBT and pTRG to construct pBT-*hcp1*, pBT-*hcp3*, pTRG-*hcp2* and pTRG-*hcp3* plasmids, respectively. Then, pBT and pTRG plasmids were paired into XL1-Blue Reporter Strain. Bacteria strains that were successfully transferred with the two corresponding plasmids were cultured at 37°C overnight. Then they were normalized to OD600 ~ 0.3 and resuspended with M9 media. Finally, 10 μL of the suspensions was spotted on dual selective screening plate and nonselective screening plate, respectively, at 30°C for 24 h.

### Statistical analyses

The data obtained were analyzed and plotted with Graphpad prism version 5.0. The comparison between the wild type strain and mutant strains was performed by student’s *t* test, and the mean ±SD from more than three independent experiments was calculated. Analysis of mouse model’s survival was performed by chi-square test using the SAS 9.0 software.

## Supplementary Material

Supplemental Material

## References

[1] Lasica AM, Ksiazek M, Madej M, et al. The type IX secretion system (T9SS): highlights and recent insights into its structure and function. Front Cell Infect Microbiol. 2015;7:215. 10.3389/fcimb.2017.00215 PMC5445135 28603700

[2] Abby SS, Rocha EPC. Identification of protein secretion systems in bacterial genomes using MacSyFinder. Sci Rep. 2016;6. DOI:10.1007/978-1-4939-7033-9_1 28667599

[3] Stefan P, Ma AT, Derek S, et al. Identification of a conserved bacterial protein secretion system in Vibrio cholerae using the Dictyostelium host model system. Proc Natl Acad Sci U S A. 2006. DOI:10.1073/pnas.0510322103 PMC1345711 16432199

[4] Macintyre DL, Miyata ST, Maya K, et al. The Vibrio cholerae type VI secretion system displays antimicrobial properties. Proc Natl Acad Sci U S A. 2010;107:19520–19524. 10.1073/pnas.1012931107 PMC2984155 20974937

[5] Sana TG, Flaugnatti N, Lugo KA, et al. Salmonella Typhimurium utilizes a T6SS-mediated antibacterial weapon to establish in the host gut. Proc Natl Acad Sci U S A. 2016;113:E5044-E5051. 27503894 10.1073/pnas.1608858113 PMC5003274

[6] Sandra S, Hood RD, Mougous JD. What is type VI secretion doing in all those bugs? Trends Microbiol. 2010. DOI:10.1016/j.tim.2010.09.001 PMC2991376 20961764

[7] Boyer F, Fichant G, Berthod J, et al. Dissecting the bacterial type VI secretion system by a genome wide in silico analysis: what can be learned from available microbial genomic resources? BMC Genomics. 2009;10:104. 10.1186/1471-2164-10-104 PMC2660368 19284603

[8] Kirk MD, Pires SM, Black RE, et al. World Health Organization estimates of the global and regional disease burden of 22 foodborne bacterial, protozoal, and viral diseases, 2010: a data synthesis. PLoS Med. 2015. DOI:10.1371/journal.pmed.1001921 PMC4668831 26633831

[9] Havelaar AH, Kirk MD, Torgerson PR, et al. World Health Organization global estimates and regional comparisons of the burden of foodborne disease in 2010. PLoS Med. 2015;12:e1001923. 10.1371/journal.pmed.1001923 PMC4668832 26633896

[10] Fields PI, Swanson RV, Haidaris CG, et al. Mutants of *Salmonella typhimurium* that cannot survive within the macrophage are avirulent. Proc Natl Acad Sci U S A. 1986;83:5189–5193. 10.1073/pnas.83.14.5189 PMC323916 3523484

[11] Herrero-Fresno A, Olsen JE. *Salmonella Typhimurium* metabolism affects virulence in the host – a mini-review. Food Microbiol. 2018;71:98. 29366476 10.1016/j.fm.2017.04.016

[12] Mulder DT, Cooper CA, Coombes BK. Type VI secretion system-associated gene clusters contribute to pathogenesis of *Salmonella enterica serovar Typhimurium*. Infect Immun. 2012;80:1996–2007. 10.1128/IAI.06205-11 PMC3370595 22493086

[13] Pezoa D, Blondel CJ, Silva CA, et al. Only one of the two type VI secretion systems encoded in the *Salmonella enterica serotype Dublin* genome is involved in colonization of the avian and murine hosts. Vet Res. 2014;45:2. 10.1186/1297-9716-45-2 PMC3899618 24405577

[14] Holden DW. Trafficking of the Salmonella vacuole in macrophages. Traffic. 2010. DOI:10.1034/j.1600-0854.2002.030301.x 11886586

[15] Riquelme S, Varas M, Valenzuela C, et al. Relevant genes linked to virulence are required for salmonellatyphimurium to survive intracellularly in the social amoeba dictyostelium discoideum. Front Microbiol. 2016;7. DOI:10.3389/fmicb.2016.01305 PMC4993766 27602025

[16] Smriti V, Srikanth CV. Understanding the complexities of *Salmonella*-host crosstalk as revealed by in vivo model organisms. IUBMB Life. 2015. DOI:10.1002/iub.1393 26179888

[17] Ji L, Ji-Tao G, Yong-Guo L, et al. The type VI secretion system gene cluster of Salmonella typhimurium: required for full virulence in mice. J Basic Microbiol. 2013. DOI:10.1002/jobm.201200047 22961625

[18] Pezoa D, Yang HJ, Blondel CJ, et al. The type VI secretion system encoded in SPI-6 plays a role in gastrointestinal colonization and systemic spread of *Salmonella enterica serovar Typhimurium* in the chicken. PLoS One. 2013;8:e63917. 10.1371/journal.pone.0063917 PMC3653874 23691117

[19] Journet L, Cascales E. The type VI secretion system in *Escherichia coli* and related species. Ecosal Plus. 2016;7. DOI:10.1128/ecosalplus.ESP-0009-2015 PMC11575709 27223818

[20] Osipiuk J, Xu X, Cui H, et al. Crystal structure of secretory protein Hcp3 from *Pseudomonas aeruginosa*. J Struct Funct Genomics. 2011;12:21–26. 10.1007/s10969-011-9107-1 PMC3365581 21476004

[21] Brunet YR, Henin J, Celia H, et al. Type VI secretion and bacteriophage tail tubes share a common assembly pathway. EMBO Rep. 2014. DOI:10.1002/embr.201337936 PMC3989698 24488256

[22] Silverman J, Agnello D, Zheng H, et al. Haemolysin coregulated protein is an exported receptor and chaperone of type VI secretion substrates. Mol Cell. 2013;51:584–593. 10.1016/j.molcel.2013.07.025 PMC3844553 23954347

[23] Lin QP, Gao ZQ, Geng Z, et al. Crystal structure of the putative cytoplasmic protein STM0279 (Hcp2) from Salmonella typhimurium. Acta Crystallogr F Struct Biol Commun. 2017;73:463–468. 10.1107/S2053230X17010512 PMC5544003 28777089

[24] Stefan P, Ma AT, Revel AT, et al. Type VI secretion system translocates a phage tail spike-like protein into target cells where it cross-links actin. Proc Natl Acad Sci U S A. 2007. DOI:10.1073/pnas.0706532104 PMC2000545 17873062

[25] Gallique M, Decoin V, Barbey C, et al. Contribution of the pseudomonas fluorescens MFE01 Type VI secretion system to biofilm formation. PLoS One. 2017;12:e0170770. 10.1371/journal.pone.0170770 PMC5256989 28114423

[26] Andersson JA, Sha J, Erova TE, et al. Identification of new virulence factors and vaccine candidates for yersinia pestis. Front Cell Infect Microbiol. 2017;7:448. 29090192 10.3389/fcimb.2017.00448 PMC5650977

[27] Decoin V, Gallique M, Barbey C, et al. Pseudomonas fluorescens type 6 secretion system is related to mucoidy, motility and bacterial competition. BMC Microbiol. 2015;15. DOI:10.1186/s12866-015-0405-9 PMC4379610 25886496

[28] Wang N, Liu J, Pang M, et al. Diverse roles of Hcp family proteins in the environmental fitness and pathogenicity of Aeromonas hydrophila Chinese epidemic strain NJ-35. Appl Microbiol Biotechnol. 2018. DOI:10.1007/s00253-018-9116-0 29862449

[29] Peng Y, Wang X, Shou J, et al. Roles of Hcp family proteins in the pathogenesis of the porcine extraintestinal pathogenic *Escherichia coli* type VI secretion system. Sci Rep. 2016. DOI:10.1038/srep26816 PMC4882540 27229766

[30] Wang J, Zhihui Z, Fang H, et al. The role of the type VI secretion system vgrG gene in the virulence and antimicrobial resistance of *Acinetobacter baumannii* ATCC 19606. PLoS One. 2018. DOI:10.1371/journal.pone.0192288 PMC5796710 29394284

[31] Tamang MD, Gurung M, Nam HM, et al. Antimicrobial susceptibility and virulence characteristics of *Salmonella enterica Typhimurium* isolates from healthy and diseased pigs in Korea. J Food Prot. 2014;77:1481–1486. 10.4315/0362-028X.JFP-14-084 25198838

[32] Schmitt CK, Ikeda JS, Darnell SC, et al. Absence of all components of the flagellar export and synthesis machinery differentially alters virulence of *Salmonella enterica serovar Typhimurium* in models of typhoid fever, survival in macrophages, tissue culture invasiveness, and calf enterocolitis. Infect Immun. 2001;69:5619–5625. 10.1128/IAI.69.9.5619-5625.2001 PMC98677 11500437

[33] Guan J, Xiao X, Xu S, et al. Roles of RpoS in Yersinia pseudotuberculosis stress survival, motility, biofilm formation and type VI secretion system expression. J Microbiol. 2015;53:633–642. 10.1007/s12275-015-0099-6 26310305

[34] Storey D, McNally A, Åstrand M, et al. Klebsiella pneumoniae type VI secretion system-mediated microbial competition is PhoPQ controlled and reactive oxygen species dependent. PLoS Pathog. 2020;16:e1007969. 10.1371/journal.ppat.1007969 PMC7108748 32191774

[35] Weber B, Hasic M, Chen C, et al. Type VI secretion modulates quorum sensing and stress response in Vibrio anguillarum. Environ Microbiol. 2009;11:3018–3028. 10.1111/j.1462-2920.2009.02005.x 19624706

[36] Aubert DF, Flannagan RS, Valvano MA. A novel sensor kinase-response regulator hybrid controls biofilm formation and type VI secretion system activity in *Burkholderia cenocepacia*. Infect Immun. 2008;76:1979–1991. 10.1128/IAI.01338-07 PMC2346693 18316384

[37] Iyoda S, Kamidoi T, Hirose K, et al. A flagellar gene fliZ regulates the expression of invasion genes and virulence phenotype in *Salmonella enterica serovar Typhimurium*. Microb Pathog. 2001;30:81–90. 10.1006/mpat.2000.0409 11162188

[38] Fernanda DP, Gerson N, Alline P, et al. The type VI secretion system plays a role in type 1 fimbria expression and pathogenesis of an avian pathogenic *Escherichia coli* strain. Infect Immun. 2010. DOI:10.1128/IAI.00531-10 PMC2981326 20855516

[39] Brunet YR, Khodr A, Logger L, et al. Silencing of the salmonella pathogenicity island 6-encoded type VI secretion system limits salmonella enterica serovar typhimurium interbacterial killing. Infect Immun. 2015;83:2738–2750. 25916986 10.1128/IAI.00198-15 PMC4468533

[40] Chakib M, Friederike H, Henry H, et al. Conflicting needs for a *Salmonella* hypervirulence gene in host and non-host environments. Mol Microbiol. 2010. DOI:10.1046/j.1365-2958.2002.03070.x 12180921

[41] Parsons DA, Heffron F. sciS, an icmF homolog in Salmonella enterica serovar Typhimurium, limits intracellular replication and decreases virulence. Infect Immun. 2005;73:4338–4345. 10.1128/IAI.73.7.4338-4345.2005 PMC1168621 15972528

[42] Ho TD, Slauch JM. Characterization of grvA, an antivirulence gene on the gifsy-2 phage in Salmonella enterica serovar typhimurium. J Bacteriol. 2001. DOI:10.1128/JB.183.2.611-620.2001 PMC94917 11133955

[43] Saliba A-E, Lei L, Westermann AJ, et al. Single-cell RNA-seq ties macrophage polarization to growth rate of intracellular Salmonella. Nat Microbiol. 2016. DOI:10.1038/nmicrobiol.2016.206 27841856

[44] Grant AJ, Morgan FJE, McKinley TJ, et al. Attenuated salmonella typhimurium lacking the pathogenicity island-2 type 3 secretion system grow to high bacterial numbers inside phagocytes in mice. PLoS Pathog. 2012;8(12):e1003070. 23236281 10.1371/journal.ppat.1003070 PMC3516571

[45] Petra F, Kowal AS, Pascale G, et al. Protocols for growth and development of *Dictyostelium discoideum*. Nat Protoc. 2007. DOI:10.1038/nprot.2007.178 17545967

[46] Datsenko KA, Wanner BL. One-step inactivation of chromosomal genes in *Escherichia coli* K-12 using PCR products. Proc Natl Acad Sci U S A. 2000;97:6640–6645. 10.1073/pnas.120163297 PMC18686 10829079

[47] Zhai YJ, Huang H, Liu J, et al. CpxR overexpression increases the susceptibility of acrB and cpxR double-deleted Salmonella enterica serovar Typhimurium to colistin. J Antimicrob Chemother. 2018;73:3016–3024. 10.1093/jac/dky320 30107570

