# Supplementary material for: Roles of the Hcp family proteins in the pathogenicity of Salmonella typhimurium 14028s
Source: Virulence. 2020 Dec 10;11(1):1716–26. doi: 10.1080/21505594.2020.1854538 (PMC7733977; doi:10.1080/21505594.2020.1854538)
Supplement: Supplemental Material [file KVIR_A_1854538_SM1415.zip › Additional file 2 Figure S2.docx]

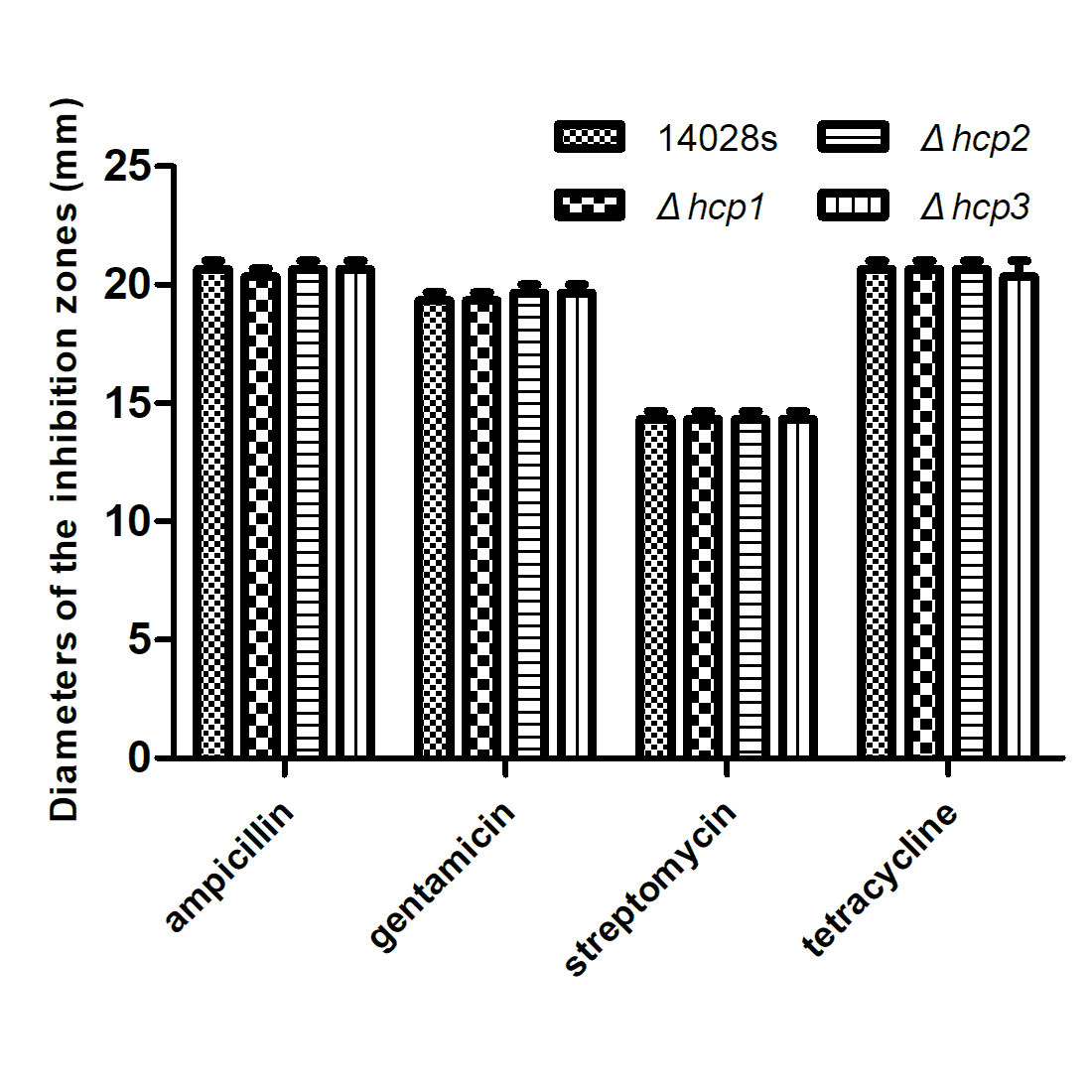


**Figure S2** Effects of hcp mutations on the MICs of antimicrobials by determining the diameters of the inhibition zones (mm) caused by four different susceptibility paper disc (ampicillin, gentamicin, streptomycin and tetracycline). Significant differences were defined by P<0.05(*), P<0.01(**) and P<0.001(***) compared to the wild type strain 14028s.
