# Supplementary material for: Roles of the Hcp family proteins in the pathogenicity of Salmonella typhimurium 14028s
Source: Virulence. 2020 Dec 10;11(1):1716–26. doi: 10.1080/21505594.2020.1854538 (PMC7733977; doi:10.1080/21505594.2020.1854538)
Supplement: Supplemental Material [file KVIR_A_1854538_SM1415.zip › Additional file 3 Figure S3.docx]

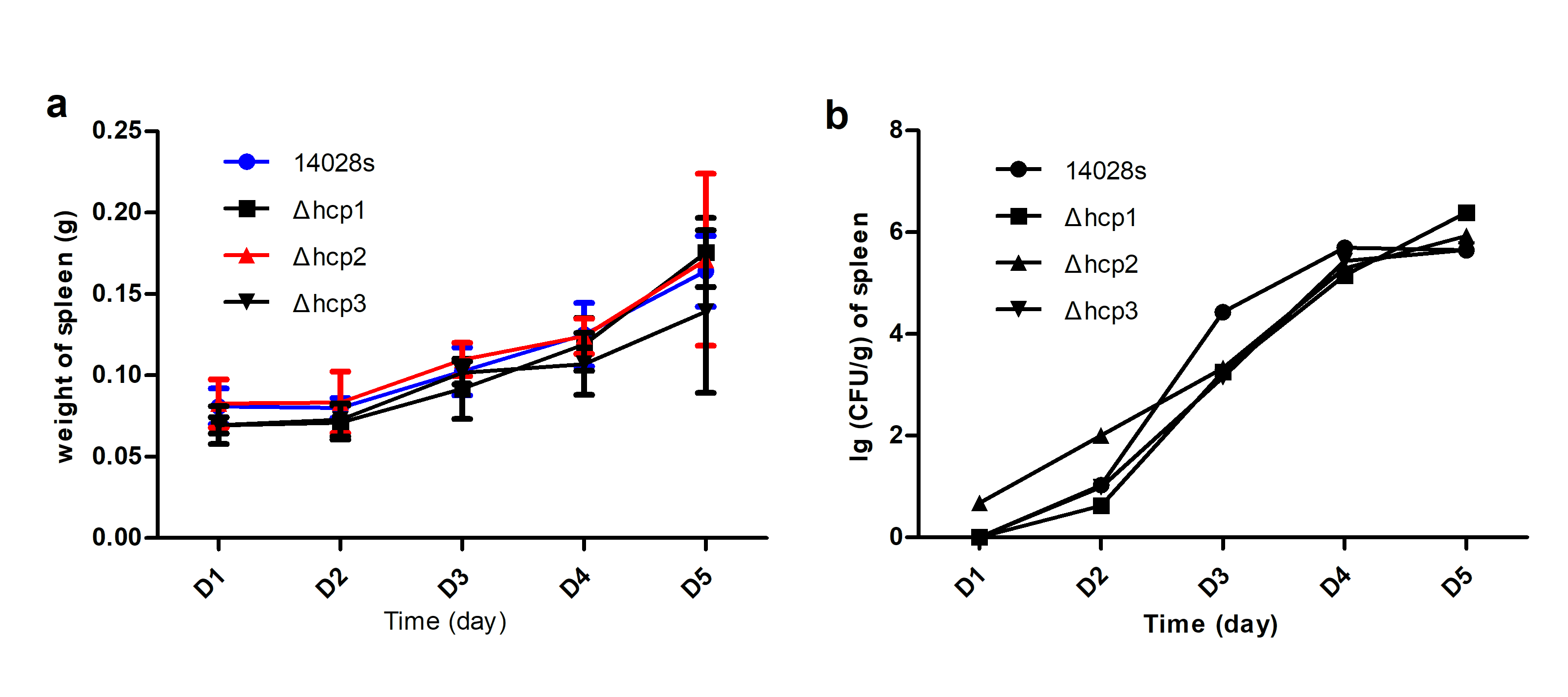


**Figure S3** The weight (a) and the number of bacteria (b) of spleen when challenged mice with 200μL bacterial suspensions normalized to 10^6^ CFU/mL. Error bars represent the SDs. Significant differences were defined by P<0.05(*), P<0.01(**) and P<0.001(***) compared to the wild type strain 14028s.
