# Supplementary material for: Roles of the Hcp family proteins in the pathogenicity of Salmonella typhimurium 14028s
Source: Virulence. 2020 Dec 10;11(1):1716–26. doi: 10.1080/21505594.2020.1854538 (PMC7733977; doi:10.1080/21505594.2020.1854538)
Supplement: Supplemental Material [file KVIR_A_1854538_SM1415.zip › Additional file 5 Table S1.docx]

| **Table S1 Bacteria strains and plasmids used in this study** | | |
| --- | --- | --- |
| Strains or Plasmids | Features | Source |
| *S.*Typhimurium | | |
| 14028s | Wild-type strain | (Jarvik et al. 2010) |
| 14028s Δ*hcp1* | 14028s STM14_0324 gene knock-out mutant, Cam^R^ | Laboratory collection |
| 14028s Δ*hcp2* | 14028s STM14_0327 gene knock-out mutant, Cam^R^ | Laboratory collection |
| 14028s Δ *hcp3* | 14028s STM14_3785 gene knock-out mutant, Kana^R^ | This study |
| 14028s Δ*hcp1*-pTrc99a | *hcp*1 complemented strain::Cam^R^,Amp^R^ | This study |
| 14028s Δ*hcp2*-pTrc99a | *hcp*2 complemented strain::Cam^R^,Amp^R^ | This study |
| 14028s Δ*hcp3*-pTrc99a | *hcp*3 complemented strain::Amp^R^ | This study |
| Escherichia coli | | |
| B/r | Wild-type strain | DictyBase |
| JM109 | Wild-type strain | (Yanisch-Perron et al. 1985) |
| XL1- BlueReporter Strain | Wild-type strain | Stratagene |
| *Dictyosteliumdiscoideum* | | |
| AX4 DBS0302402 | Wild-type strain | DictyBase |
| Plasmids | | |
| pKD46 | Amp^R^ | (Uzzau et al. 2001) |
| pKD4 | Kan^R^, Amp^R^ | (Uzzau et al. 2001) |
| pCP20 | Amp^R^ | (Uzzau et al. 2001) |
| pTrc99a | Amp^R^ | (Amann et al. 1988) |
| pBT | Cam^R^ | Stratagene |
| pTRG | Tc^R^ | Stratagene |

**References**

Amann E, Ochs B, Abel K-J. Tightly regulated tac promoter vectors useful for the expression of unfused and fused proteins in *Escherichia coli*. Gene. 1988. doi: 10.1016/0378-1119(88)90440-4

Jarvik T, Smillie C, Groisman EA, Ochman H. Short-Term Signatures of Evolutionary Change in the *Salmonella enterica Serovar Typhimurium* 14028 Genome. J Bacteriol. 2010. doi: 10.1128/JB.01233-09

Uzzau S, Figueroa-Bossi N, Rubino S, Bossi L. Epitope tagging of chromosomal genes in Salmonella. Proc Natl Acad Sci U S A. 2001. doi: 10.1073/pnas.261348198

Yanisch-Perron C, Vieira J, Messing J. Improved M13 phage cloning vectors and host strains: nucleotide sequences of the M13mpl8 and pUC19 vectors. Gene. 1985. doi:10.1016/0378-1119(85)90120-9
