# Supplementary material for: Roles of the Hcp family proteins in the pathogenicity of Salmonella typhimurium 14028s
Source: Virulence. 2020 Dec 10;11(1):1716–26. doi: 10.1080/21505594.2020.1854538 (PMC7733977; doi:10.1080/21505594.2020.1854538)
Supplement: Supplemental Material [file KVIR_A_1854538_SM1415.zip › Additional file 6 Table S2.docx]

**Table S2. Primers used in this study**

| Primer | Nucleotide sequence (5′→3′) | The amplified fragment or the utilization |
| --- | --- | --- |
| *hcp3*-F  *hcp3*-R | GCCGCCGGTATTCGTCTGGCTGCTGAACTGCCACCTGATCAACAAGGAGATAAATC GTGTAGGCTGGAGCTGCTTC  TATCCCCTCAACCAAGAGCCTGTCGAATAGCGCGACCGGAATGGTTGGTGTCATCA CATATGAATATCCTCCTTAG | DNA fragment of 1581-bp of the kanamycin resistance ORF sequence and coding sequence fusing with *hcp3* upstream and downstream encoded sequence. Used for construction of *hcp3* deletion mutant. |
| *hcp1-*PCF  *hcp1-*PCR | TGACGCAACCAATACCTGCT  CAGCCAGACATAACATCTGGC | Used for PCR confirming of *hcp1* deletion mutant. |
| *hcp2-*PCF  *hcp2-*PCR | GCATACGTTCTCTTGCTCTG  TACGCCATTCATTCACTGTA | Used for PCR confirming of *hcp2* deletion mutant. |
| *hcp3-*PCF  *hcp3-*PCR | TTGCGTCGTACCAATTCATG  ATAAGAGTCACAAGCGCTGG | Used for PCR confirming of *hcp3* deletion mutant. |
| *hcp1-*cF  *hcp1-*cR | CGGGATCCAACTTAATAAGGATATAAAA  TTAAGCTTTCTGGCCGGAAAAACAGCCG | Used for construction of *Δhcp1* complement |
| *hcp2-*cF  *hcp2-*cR | GGGGATCCTAGTTAAAAGGATAGTAGAT  TTAAGCTTTCTGGCCGGAAAAACAGCCG | Used for construction of *Δhcp2* complement |
| *hcp3-*cF  *hcp3-*cR | ACGGATCCCGGAATGGTTGGTGTCATCA  TCAAGCTTGATCAACAAGGAGATAAATC | Used for construction of *Δhcp3* complement |
| *hcp1*-qF  *hcp1*-qR | CGCTGGAGTACCTCAAGTATACC  AAGGAGAGCTCCACCGTTTC | 109-bp internal fragment of *hcp1*, used for qRT-PCR. |
| *hcp2*-qF  *hcp2*-qR | CGCTGGAGTACCTCAAGTACACT  AAGGAGAGTTCAATTGATTC | 109-bp internal fragment of *hcp2*, used for qRT-PCR. |
| *hcp3*-qF  *hcp3*-qR | AGCACAGGCGTGGCGAGGTC  GCAATGCAGGTAAATAACGA | 107-bp internal fragment of *hcp3*, used for qRT-PCR. |
| *fimH*-qF  *fimH*-qR | GCAGGACTCAAAGCTGGTTT  CGGTAGAGGTCGTCACATAGA | 107-bp internal fragment of *fimH*, used for qRT-PCR. |
| *fljb*-qF  *fljb*-qR | ATGGCACAAGTAATCAACAC  GCAGACCAGAAGACAGAC | 109-bp internal fragment of *fljb*, used for qRT-PCR. |
| *flic*-qF  *flic*-qR | GAAGCAGATCAACTCTCAGA  ATCGTAGTATCGGCATATCC | 108-bp internal fragment of *flic*, used for qRT-PCR. |
| *flia*-qF  *flia*-qR | GCGGAGTATCGTCAGATG  ATGTTCTTCAGTCACCAGTT | 108-bp internal fragment of *flia*, used for qRT-PCR. |
| *flhd*-qF  *flhd*-qR | TTGACGATCATCAGACGAT  TCCACTTCATTGAGCAGAC | 109-bp internal fragment of *flhd*, used for qRT-PCR. |
| *rpoS*-qF  *rpoS*-qR | CGTATGTTGAGAAGCGGAACC  CAATCGTGGACTGGCGTTG | 108-bp internal fragment of *rpoS*, used for qRT-PCR. |
| 16s-F  16s-R | CGGTGGAGCATGTGGTTTAA  AAGGCACAAATCCATCTCTG | 109-bp internal fragment of the 16S rDNA sequence, used for qRT-PCR. |
| pBT-Hcp1F  pBT-Hcp1R | CGGAATTCATGGCTTATGACATTTTTTTG  CG GGATCC ATGGTGATGGTGATGATGTTAAATTTCTTTGTTGGCCT | Used for bacterial two-hybrid assay. |
| pBT-Hcp3F  pBT-Hcp3R | CGGAATTCTCATTTCCCTATCTGGTTCA  CGGGATCCATGGTGATGGTGATGATG ATGGATGCGATTTTTTTAAA | Used for bacterial two-hybrid assay. |
| pTRG-Hcp2F  pTRG-Hcp2R | CGGGATCCATGTCTTATGACATTTTTCT  CGGAATTCATGGTGATGGTGATGATG TTAAATTTCTTTGTTGGCC | Used for bacterial two-hybrid assay. |
| pTRG-Hcp3F  pTRG-Hcp3R | CGGGATCCTCATTTCCCTATCTGGTTCA  CGGAATTCATGGTGATGGTGATGATG ATGGATGCGATTTTTTTAAA | Used for bacterial two-hybrid assay. |
