# Supplementary figures and images for: Roles of the Hcp family proteins in the pathogenicity of Salmonella typhimurium 14028s
Source: Virulence. 2020 Dec 10;11(1):1716–26. doi: 10.1080/21505594.2020.1854538 (PMC7733977; doi:10.1080/21505594.2020.1854538)

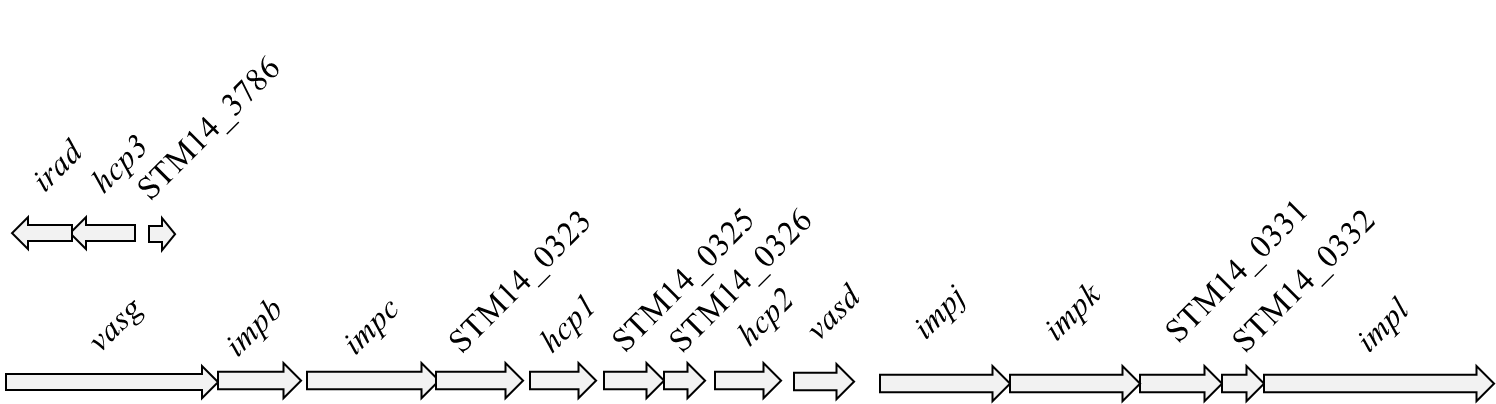


**Figure S1** Genetic organization of the *hcp*s loci in *Salmonella typhimurium* 14028s.

Supplement: Supplemental Material [file KVIR_A_1854538_SM1415.zip › Additional file 1 Figure S1.docx]
